# Supplementary material for: Structural mechanisms of TRPV6 inhibition by ruthenium red and econazole
Source: Nat Commun. 2021 Nov 1;12:6284. doi: 10.1038/s41467-021-26608-x (PMC8560856; doi:10.1038/s41467-021-26608-x)
Supplement: Supplementary file 1 — Supplementary Information [file 41467_2021_26608_MOESM1_ESM.pdf]

Supplementary Information

Structural mechanisms of TRPV6 inhibition by ruthenium red and econazole

Arthur Neuberger[1,2], Kirill D. Nadezhdin[1,2] and Alexander I. Sobolevsky[1*]

[1] Department of Biochemistry and Molecular Biophysics, Columbia University, New York, New York, USA

[2] These authors contributed equally to this work

* Correspondence and requests for materials should be addressed to A.I.S. (Email: as4005@cumc.columbia.edu; Tel: 212-305-4249)

This PDF file includes:

Supplementary Figures 1-5

Supplementary Tables 1-2

References

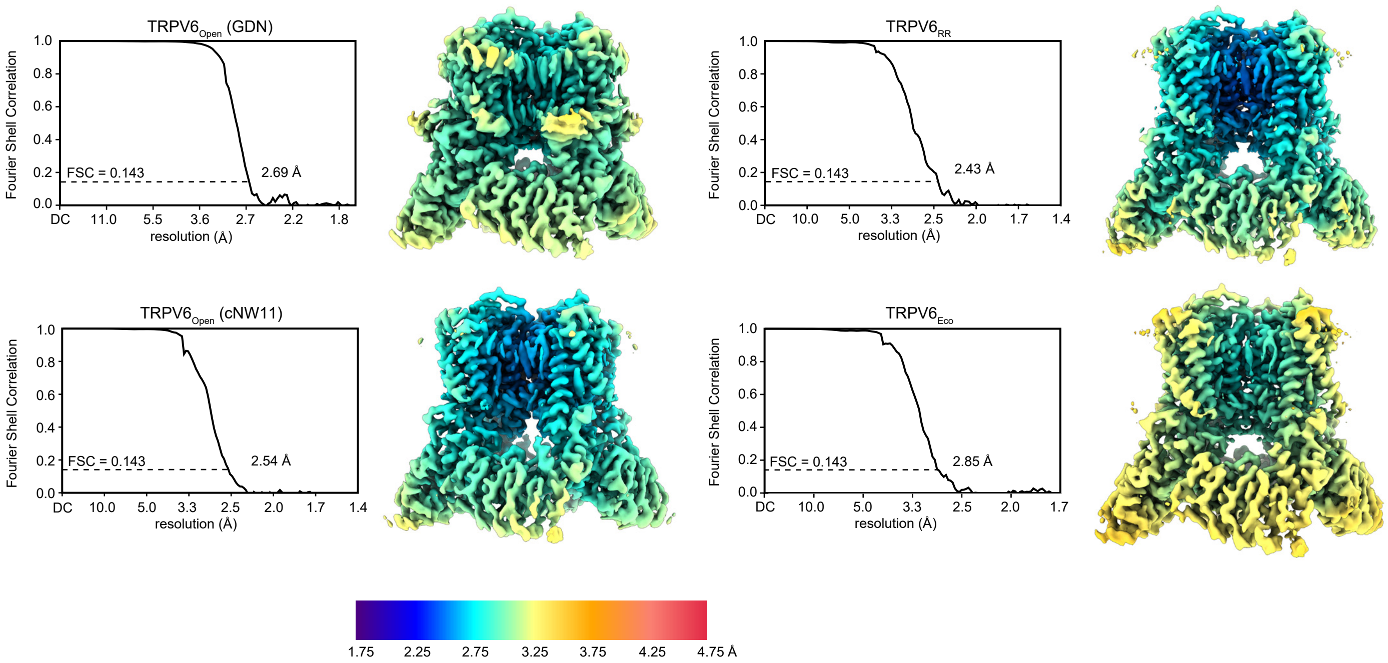

Supplementary Fig. 1. Characteristics of TRPV6 cryo-EM reconstructions. Plots show corrected FSC curves calculated between half maps, with the overall resolution estimated using the FSC = 0.143 criterion. Cryo-EM maps are colored according to the local resolution estimation in RELION.

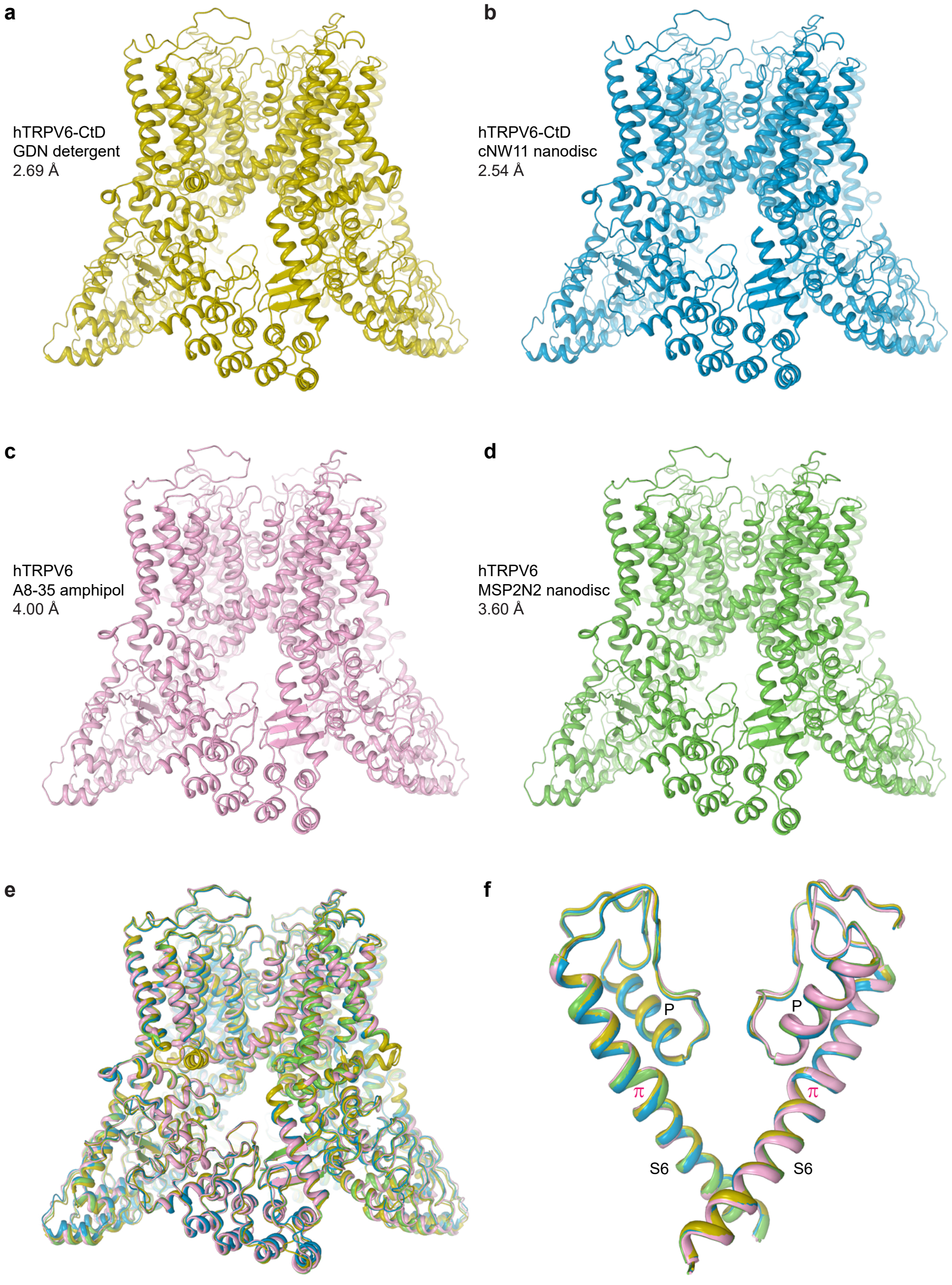

Supplementary Fig. 2. Comparison of human TRPV6 open-state structures. a-d, Cryo-EM open-state (apo) structures of hTRPV6-CtD in GDN detergent (**a**, olive), hTRPV6-CtD in cNW11 nanodisc (**b**, blue), hTRPV6 in A8-35 amphipol (**c**, pink, PDB ID: 6BO9), and hTRPV6 in MSP2N2 nanodisc (**d**, green, PDB ID: 6BO8) viewed parallel to membrane. **e,** Superposition of structures shown in **a-d**. **f,** Close-up view of the pore-forming domains in superposition shown in **e**. Only two of four subunits are shown, with the front and back subunits omitted for clarity. The secondary structure elements and the region that undergoes α-to-π transition are labelled.

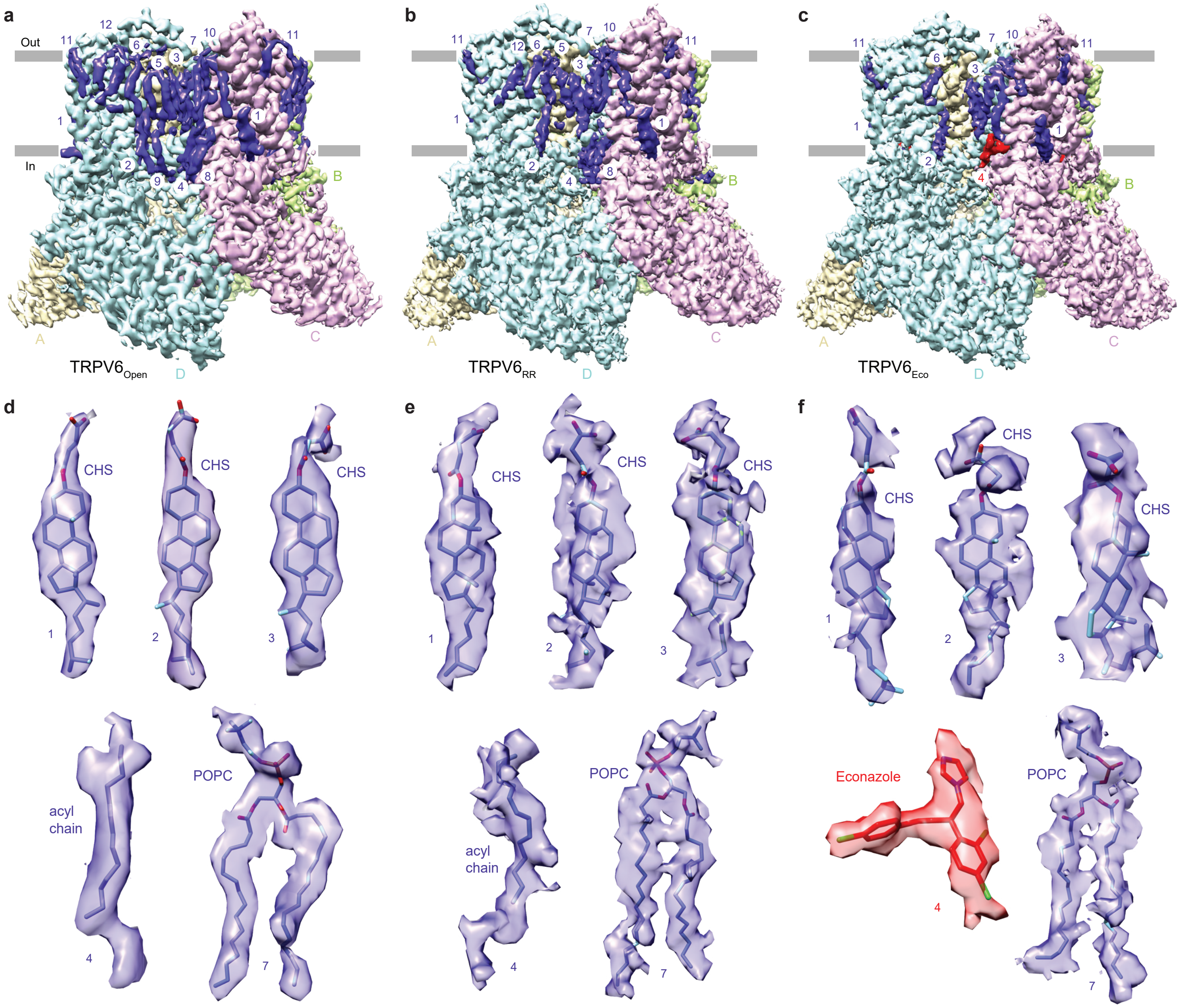

Supplementary Fig. 3. Lipid densities. a-c, Cryo-EM structures of TRPV6 in the open (apo) state (**a**), in complex with RR (**b**) or econazole (**c**) viewed parallel to the membrane, with each of the four subunits colored differently, lipids in purple and inhibitors in red. Lipid binding sites, which are identical between individual TRPV6 subunits, are numbered. **d-f,** Close-up views of semi-transparent cryo-EM density for sites 1-4 and 7 in the open (**d**), RR-bound (**e**) and econazole-bound (**f**) structures fitted with molecules of cholesteryl hemisuccinate (CHS), acyl chains, POPC or econazole shown as sticks.

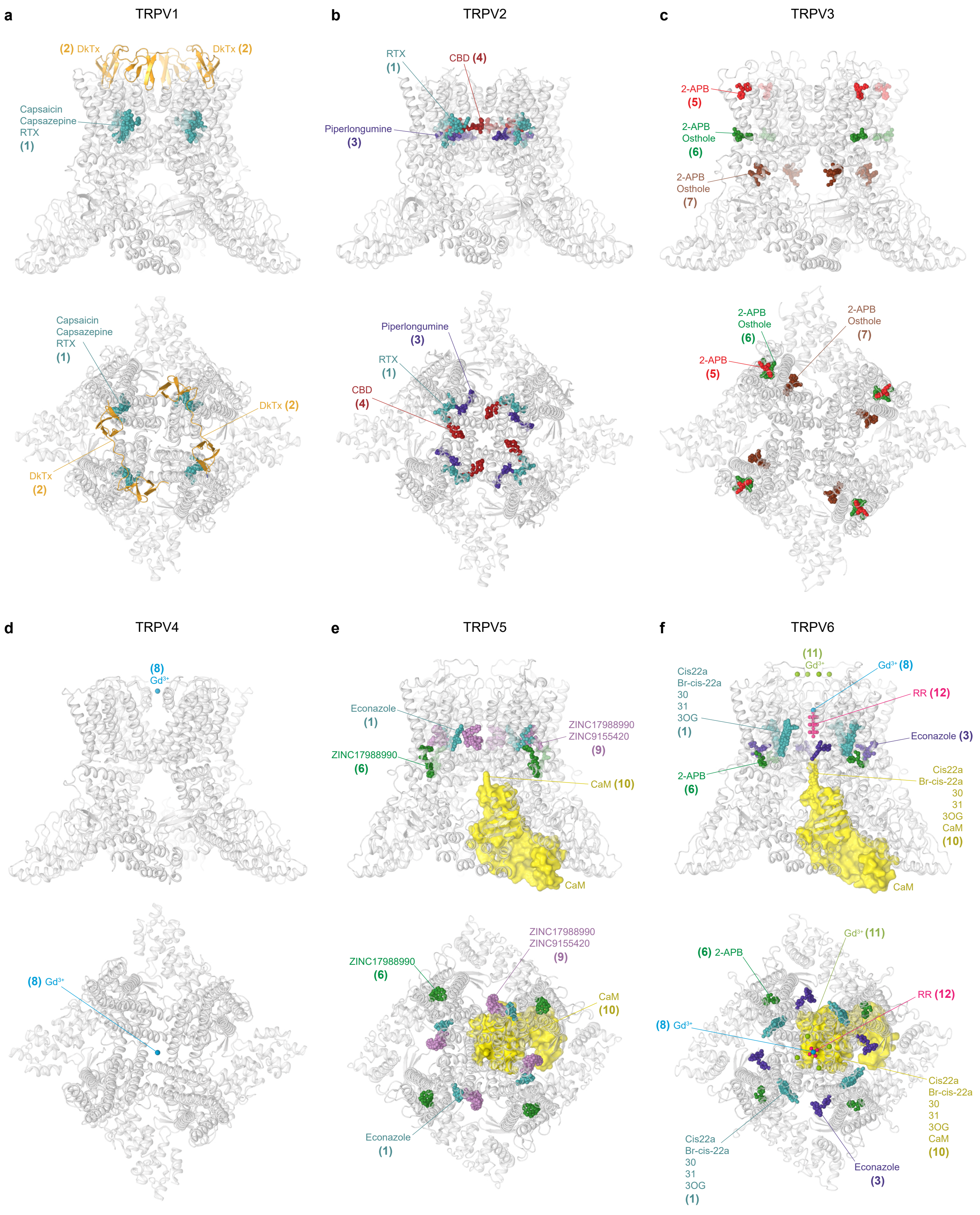

a
TRPV1
(2) DkTx
DkTx (2)
Capsaicin
Capsazepine
RTX
(1)
DkTx (2)
DkTx
(2)
b
TRPV2
RTX
(1)
CBD (4)
Piperlongumine
(3)
c
TRPV3
2-APB
(5)
2-APB
Osthole
(6)
2-APB
Osthole
(7)
d
TRPV4
(8)
Gd³⁺
(8) Gd³⁺
e
TRPV5
Econazole
(1)
ZINC17988990
ZINC9155420
(9)
ZINC17988990
(6)
CaM (10)
CaM
f
TRPV6
(11)
Gd³⁺
Gd³⁺ (8)
Cis22a
Br-cis-22a
30
31
3OG
(1)
RR (12)
Econazole (3)
2-APB
(6)
Cis22a
Br-cis-22a
30
31
3OG
CaM
(10)
CaM
(6) 2-APB
Gd³⁺ (11)
RR (12)
(8) Gd³⁺
Econazole
(3)

Supplementary Fig. 4. Binding sites in the vanilloid subfamily of TRP channels. Grey ribbons show structures of (**a**) TRPV1 in complex with agonist capsaicin (PDB ID: 7LR0)[1] superposed with agonists RTX and DkTx (PDB ID: 5IRX)[2] and antagonist capsazepine (PDB ID: 5IS0)[2], (**b**) TRPV2 in complex with agonist cannabidiol (CBD, PDB ID: 6U88)[3] superposed with agonist RTX (PDB ID: 6BWJ)[4] and antagonist piperlongumine (PDB ID: 6WKN)[5], (**c**) TRPV3 in complex with agonist 2-APB (PDB ID: 6DVZ)[6] superposed with competitive antagonist osthole (PDB ID: 7RAS)[7], (**d**) TRPV4 in complex with the channel blocker Gd^{3+} (PDB ID: 6C8H)[8], (**e**) TRPV5 in complex with antagonist ZINC 17988990 (PDB ID: 6PBE)[9] superposed with antagonists ZINC 9155420 (PDB ID: 6PBF)[9] and econazole (PDB ID: 6B5V)[10], and inactivator CaM (PDB ID: 6O20)[11] and (**f**) TRPV6 in complex with the channel blocker RR (this study) superposed with another channel blocker Gd^{3+} (PDB ID: 5WOA)[12] and antagonists 2-APB (PDB ID: 6D7T)[13], econazole (this study), cis-22a (PDB ID: 7K4B)[14], Br-cis-22a (PDB ID: 7K4C)[14], 30 (PDB ID: 7K4E)[14], 31 (PDB ID: 7K4F)[14] and 3OG (PDB ID: 7K4D)[14], and inactivator CaM (PDB ID: 6E2F)[15]. Different binding sites are emphasized by numbering and showing the corresponding ligands in distinct colors.

```
         1                                                                                                  100
hTRPV6    (1) -----------------------------------------------------------------------------------------MGLSLPKEKG-
hTRPV5    (1) -----------------------------------------------------------------------------------------MGGFLPKAEG-
hTRPV4    (1) MADSSEGPRAGPGEVAELPGDESGTPGGEAFPLSSLANLFEGEDGSLSPSPADASRPAGPGDGRPNLRMKFQGAFRKGVPN-PIDLLESTLYESSVVPGP
hTRPV3    (1) ---------------------------MKAHPKEMVPLMGKRVAAPSGNP--AILPEKRPAEITPTKKSAHFFLEIEGFEPNPTVAKTSPPVFSKPMDSN
hTRPV2    (1) ----------------------------------------------------MTSPSSSP----------VFRLETLDGG--QEDGSEADRGKLDFGSG-
hTRPV1    (1) ---------------------------MKKWSSTDLGAAADPLQKDTCPDPLDGDPNSRPPPAKP--QLSTAKSRTRLFG--KGDSEEAFPVDCPHEEGE

                                                    N-terminal helix          Ankyrin Repeat 1
         101                                                                                                200
hTRPV6   (11) ----------LILCLWS-----KFCRWFQRRES------WAQSRDEQNLLQQKRIWESPLLLAAK--DNDVQALNKLLKYEDCKVH--------QRGAMG
hTRPV5   (11) ----------PGSQLQK-----LLPSFLVREQD------WDQHLDKLHMLQQKRILESPLLRASK--ENDLSVLRQLLLDCTCDVR--------QRGALG
hTRPV4  (100) KKAPMDSLFDYGTYRHHSSDNKRWRKKIIEKQPQSPKAPAPQPPPILKVFNRPILFDIVSRGSTADLDGLLPFLLTHKKRLTDEEF--------REPSTG
hTRPV3   (72) IRQCISGNCDDMDSPQSPQDDVTETPSNPNSPS---AQLAKEEQRRKKRRLKKRIFAAVSEGCVEELVELLVELQELCRRRHDEDVPDFLMHKLTASDTG
hTRPV2   (36) -----LPPMESQFQGEDRKFAPQIRVNLNYRKG------TGASQPDPNRFDRDRLFNAVSRGVPEDLAGLPEYLSKTSKYLTDSEY--------TEGSTG
hTRPV1   (70) LDSCPTITVSPVITIQRPGDGPTGARLLSQDSV------AASTEKTLRLYDRRSIFEAVAQNNCQDLESLLLFLQKSKKHLTDNEF--------KDPETG

                   Ankyrin Repeat 2                                       Ankyrin Repeat 3
         201                                                                                                300
hTRPV6   (80) ETALHIAALYDNLEA----AMVLMEAAP-----ELVFEPMTSELYEGQTALHIAVVNQNMNLVRALLARRASVSARATGTAFRRSPC-NLIYFGEHPLSF
hTRPV5   (80) ETALHIAALYDNLEA----ALVLMEAAP-----ELVFEPTTCEAFAGQTALHIAVVNQNVNLVRALLTRRASVSARATGTAFRRSPR-NLIYFGEHPLSF
hTRPV4  (192) KTCLPKALLNLSNGRNDTIPVLLDIAERTGNMREFINSPFRDIYYRGQTALHIAIERRCKHYVELLVAQGADVHAQARGRFFQPKDEGGYFYFGELPLSL
hTRPV3  (169) KTCLMKALLNINPNTKEIVRILLAFAEENDILGRFINAEYTEEAYEGQTALNIAIERRQGDIAALLIAAGADVNAHAKGAFFNPKYQHEGFYFGETPLAL
hTRPV2  (117) KTCLMKAVLNLKDGVNACILPLLQIDRDSGNPQPLVNAQCTDDYYRGHSALHIAIEKRSLQCVKLLVENGANVHARACGRFFQKGQG-TCFYFGELPLSL
hTRPV1  (156) KTCLLKAMLNLHDGQNTTIPLLLEIARQTDSLKELVNASYTDSYYKGQTALHIAIERRNMALVTLLVENGADVQAAAHGDFFKKTKGRPGFYFGELPLSL

                 Ankyrin Repeat 4                           Ankyrin Repeat 5                        Ankyrin Repeat 6
         301                                                                                                400
hTRPV6  (170) AACVNSEEIVRLLIEHG---ADIRAQDSLGNTVLHILILQ-----PNKTFACQMYNLLLSYDRHGDHLQPLDLVPNHQGLTPFKLAGVEGNTVMFQHLMQ
hTRPV5  (170) AACVNSEEIVRLLIEHG---ADIRAQDSLGNTVLHILILQ-----PNKTFACQMYNLLLSYDGHGDHLQPLDLVPNHQGLTPFKLAGVEGNTVMFQHLMQ
hTRPV4  (292) AACTNQPHIVNYLTENPHKKADMRRQDSRGNTVLHALVAIADNTRENTKFVTKMYDLLLLKCARLFPDSNLEAVLNNDGLSPLMMAAKTGKIGIFQHIIR
hTRPV3  (269) AACTNQPEIVQLLMEHEQ--TDITSRDSRGNNILHALVTVAEDFKTQNDFVKRMYDMILLRSG----NWELETTRNNDGLTPLQLAAKMGKAEILKYILS
hTRPV2  (216) AACTKQWDVVSYLLENPHQPASLQATDSQGNTVLHALVMISDNSAENIALVTSMYDGLLQAGARLCPTVQLEDIRNLQDLTPLKLAAKEGKIEIFRHILQ
hTRPV1  (256) AACTNQLGIVKFLLQNSWQTADISARDSVGNTVLHALVEVADNTADNTKFVTSMYNEILMLGAKLHPTLKLEELTNKKGMTPLALAAGTGKIGVLAYILQ

                          β1      β2     ARD-TM Linker    LH1         LH2        Pre-S1             S1
         401                                                                                                500
hTRPV6  (262) -----------KRKHTQWTYGPLTSTLYDLTEIDSSGDEQSLLELIIT-TKKREARQILDQTPVKELVSLKWKRYGRPYFCMLGAIYLLYIICFTMCCIY
hTRPV5  (262) -----------KRRHIQWTYGPLTSILYDLTEIDSWGEELSFLELVVS-SDKREARQILEQTPVKELVSFKWNKYGRPYFCILAALYLLYMICFTTCCVY
hTRPV4  (392) REVTDEDTRHLSRKFKDWAYGPVYSSLYDLSSLDTCGEEASVLEILVYNSKIENRHEMLAVEPINELLRDKWRKFGAVSFYINVVSYLCAMVIFTLTAYY
hTRPV3  (363) REIKEKRLRSLSRKFTDWAYGPVSSSLYDLTNVDTTTDNSVLEITVYN-TNIDNRHEMLTLEPLHTLLHMKWKKFAKHMFFLSFCFYFFYNITLTLVSYY
hTRPV2  (316) REFSG--LSHLSRKFTEWCYGPVRVSLYDLASVDSCEENSVLEIIAFH-CKSPHRHRMVVLEPLNKLLQAKWDLLIP-KFFLNFLCNLIYMFIFTAVAYH
hTRPV1  (356) REIQEPECRHLSRKFTEWAYGPVHSSLYDLSCIDTCEKNSVLEVIAYSSSETPNRHDMLLVEPLNRLLQDKWDRFVKRIFYFNFLVYCLYMIIFTMAAYY

                         S1-S2 Loop                          S2                                  S3
         501                                                                                                600
hTRPV6  (350) RPLKPRTNNRTSPRDNTLLQQKLLQEAYMTPKDDIRLVGELVTVIGAIIILLVEVPDIFRMGVTRFFGQTILGGPFHVLIITYAFMVLVTMVMRLISASG
hTRPV5  (350) RPLKFRGGNRTHSRDITILQQKLLQEAYETREDIIRLVGELVSIVGAVIILLLEIPDIFRVGASRYFGKTILGGPFHVIIITYASLVLVTMVMRLTNTNG
hTRPV4  (492) QP-----------------LEGTPPYPYRTTVDYLRLAGEVITLFTGVLFFFTNIKDLFMK-KCPGVNSLFIDGSFQLLYFIYSVLVIVSAALYLAGIEA
hTRPV3  (462) RPR-----------E---EEAIPHPLALTHKMGWLQLLGRMFVLIWAMCISVKEGIAIFLLR-PSDLQSILSDAWFHFVFFIQAVLVILSVFLYLFAYKE
hTRPV2  (412) QPT---------------LKKQAAPHLKAEVGNSMLLTGHILILLGGIYLLVGQLWYFWRR--HVFIWISFIDSYFEILFLFQALLTVVSQVLCFLAIEW
hTRPV1  (456) RP-----------------VDGLPPFKMEKTGDYFRVTGEILSVLGGVYFFFRGIQYFLQR--RPSMKTLFVDSYSEMLFFLQSLFMLATVVLYFSHLKE

                      S4                           S5
         601                                                                                                700
hTRPV6  (450) EVVPMSFALVLGWCNVMYFARGFQMLGPFTIMIQKMIFGDLMRFCWLMAVVILGFASAFYIIFQTEDPEELGH----------------------FYDYP
hTRPV5  (450) EVVPMSFALVLGWCSVMYFTRGFQMLGPFTIMIQKMIFGDLMRFCWLMAVVILGFASAFYIIFQTEDPTSLGQ----------------------FYDYP
hTRPV4  (574) YLAVMVFALVLGWMNALYFTRGLKLTGTYSIMIQKILFKDLFRFLLVYLLFMIGYASALVSLLNPCANMKVCN------EDQTNCTVPTYPSCRDSETFS
hTRPV3  (547) YLACLVLAMALGWANMLYYTRGFQSMGMYSVMIQKVILHDVLKFLFVYIVFLLGFGVALASLIEKCPKDNKDCS--------------------SYGSFS
hTRPV2  (495) YLPLLVSALVLGWLNLLYYTRGFQHTGIYSVMIQKVILRDLLRFLLIYLVFLFGFAVALVSLSQEAWRPEAPTGPNATESVQPMEGQEDEGNGAQYRGIL
hTRPV1  (537) YVASMVFSLALGWTNMLYYTRGFQQMGIYAVMIEKMILRDLCRFMFVYIVFLFGFSTAVVTLIEDGKNDSLPS----ESTSHRWRGPACRPPDSSYNSLY

                  P     S.F.                    S6                         TRP Helix          C-terminal Hook
         701                                                                                                800
hTRPV6  (528) MALFSTFELFLTIIDGPANYNVDLPFMYSITYAAFAIIATLLMLNLLIAMMGDTHWRVAHERDELWRAQIVATTVMLERKLPRCLWPRS------GI-CG
hTRPV5  (528) MALFTTFELFLTVIDAPANYDVDLPFMFSIVNFAFAIIATLLMLNLFIAMMGDTHWRVAQERDELWRAQVVATTVMLERKLPRCLWPRS------GI-CG
hTRPV4  (668) TFLLDLFKLTIGMGDLEMLSSTKYPVVFIILLVTYIILTFVLLLNMLIALMGETVGQVSKESKHIWKLQWATTILDIERSFPVFLRKAFRSGEMVTVGKS
hTRPV3  (627) DAVLELFKLTIGLGDLNIQQNSKYPILFLFLLITYVILTFVLLLNMLIALMGETVENVSKESERIWRLQRARTILEFEKMLPEWLRSRFRMG-----ELC
hTRPV2  (595) EASLELFKFTIGMGELAFQEQLHFRGMVLLLLLAYVLLTYILLLNMLIALMSETVNSVATDSWSIWKLQKAISVLEMENGYWWCRKKQR-AGVMLTVGTK
hTRPV1  (633) STCLELFKFTIGMGDLEFTENYDFKAVFIILLLAYVILTYILLLNMLIALMGETVNKIAQESKNIWKLQRAITILDTEKSFLKCMRKAFRSGKLLQVGYT

                        β3
         801                                                                                                900
hTRPV6  (621) REYGLGDRWFLRVEDRQDLNRQRIQRYAQAFHTRGSEDLDKDSVEK----LELGCPFSPHLSLPMPSVSRSTSRSS--ANWERLRQGTLRRDLRGIINRG
hTRPV5  (621) CEFGLGDRWFLRVENHNDQNPLRVLRYVEVFKNSDKEDDQEHPSEKQPSGAESGTLARASLALPTSSLSRTASQSSSHRGWEILRQNTLGHLNLGLNLSE
hTRPV4  (768) SDGTPDRRWCFRVDEVNWSHWNQNLGIINEDPGKNETYQYYGFSHTVGRLR---RDRWSSVVPRVVELNKNSNPDEVVVPLDSMGNPRCDGHQQGYPRKW
hTRPV3  (722) KVAEDDFRLCLRINEVKWTEWKTHVSFLNEDPGPVRRTDFNKIQDS--------------------SRNNSKTTLNAFEEVEEFPETSV-----------
hTRPV2  (694) PDGSPDERWCFRVEEVNWASWEQTLPTLCEDP--SGAGVPRTLENPVLAS-------------PPKEDEDGASEEN-YVPVQLLQSN-------------
hTRPV1  (733) PDGKDDYRWCFRVDEVNWTTWNTNVGIINEDPG-NCEGVKRTLSFSLRSSRVSGRHWKNFALVPLLREASARDRQS-AQPEEVYLRQFSGSLKPEDAEVF

         901      911
hTRPV6  (715) LEDGESWEYQI
hTRPV5  (721) GDGEEVYHF--
hTRPV4  (865) RTDDAPL----
hTRPV3  (791) -----------
hTRPV2  (765) -----------
hTRPV1  (831) KSPAASGEK--
```

Supplementary Fig. 5. Sequence alignment of human vanilloid-subfamily TRP channels. Secondary structure elements are depicted above the sequence as cylinders (α-helices), arrows (β-strands) and lines (loops). Pink rectangle marks the selectivity filter. Cyan and green circles indicate amino acid residues involved in binding of RR and econazole, respectively.

Supplementary Table 1. Cryo-EM data collection, refinement and validation statistics

Structure	**$TRPV6_{Open}$ GDN, CHS**	**$TRPV6_{Open}$ cNW11**	**$TRPV6_{RR}$ cNW11**	**$TRPV6_{Eco}$ cNW11**
EMDB accession code	EMD-24890	EMD-24891	EMD-24892	EMD-24893
PDB accession code	PDB 7S88	PDB 7S89	PDB 7S8B	PDB 7S8C
**Data collection and processing**				
Magnification	105,000x	105,000x	105,000x	105,000x
Voltage (kV)	300	300	300	300
Electron exposure ($e^-/Å^2$)	58	60	58.5	60
Defocus range (μm)	–1.0 to –2.0	–0.8 to –2.0	–0.5 to –2.0	–0.8 to –2.0
Reported pixel size (Å)	0.855	0.826	0.800	0.860
Calibrated pixel size (Å)	0.827	0.829	0.799	0.832
Exposures (no.)	7,904	5,706	4,431	3,950
**Processing software**				
Platform software for particle picking	RELION v3.1	RELION v3.1	RELION v3.1	RELION v3.1
Motion correction	MotionCor2	MotionCor2	MotionCor2	MotionCor2
CTF estimation	Gctf v1.06	Gctf v1.06	Gctf v1.06	Gctf v1.06
Software for 2D/3D class. & refinements	RELION v3.1 cryoSPARC v3.1	RELION v3.1 cryoSPARC v3.1	RELION v3.1 cryoSPARC v3.1	RELION v3.1 cryoSPARC v3.1
Symmetry imposed	C4	C4	C4	C4
Initial particle images (no.)	3,117,068	5,057,951	997,105	1,757,266
Final particle images (no.)	276,688	153,950	49,537	41,635
Map resolution (Å) FSC 0.143	2.69	2.54	2.43	2.85
Map resolution range (Å)	1.83–6.36	2.45–3.43	2.37–3.66	2.70–4.29
**Refinement**				
Initial models used (PDB code)	7K4A	7K4A	7K4B	7K4B
Model resolution (Å) FSC threshold	2.69	2.54	2.43	2.85
Map sharpening *B* factor ($Å^2$)	–117	–61	–42	–59
**Model composition**				
Non-hydrogen atoms	22,201	20,425	21,105	20,562
Protein residues	2,456	2,376	2,448	2,448
Ligands	65	45	46	34
Water	144	88	0	0
B factors ($Å^2$)				
Protein	54.05	30.26	42.17	43.98
Ligand	58.74	15.20	8.63	16.48
Water	42.18	23.77		
R.m.s. deviations				
Bond lengths (Å)	0.010	0.011	0.010	0.010
Bond angles (°)	1.450	1.493	1.599	1.438
**Validation**				
MolProbity score	1.80	1.73	1.81	1.59
Clashscore	8.89	8.05	8.77	7.71
Poor rotamers (%)	1.31	1.74	0.56	0.75
Ramachandran plot				
Favoured (%)	96.41	97.46	95.12	97.05

Supplementary Table 2. Primers for mutagenesis

Primer name	Mutation	Primer sequence
hV6-F472A-F	F472A	5’-ttcgccaggggagcccagatg-3’
hV6-F472A-R	F472A	5’-tcccctggcgaagtacatcacg-3’
hV6- L475A-F	L475A	5’-ggattccagatggcgggcc-3’
hV6- L475A -R	L475A	5’-catctggaatcccctggcgaag-3’
hV6- W495A-F	W495A	5’-atgaggttttgtgcgctcatgg-3’
hV6- W495A-R	W495A	5’-acaaaacctcatcaagtctccg-3’
hV6- T539A-F	T539A	5’-gagctgttcctggcaattattgacg-3’
hV6- T539A-R	T539A	5’-caggaacagctcaaacgtggag-3’

Supplementary References

1 Nadezhdin, K. D. *et al.* Extracellular cap domain is an essential component of the TRPV1 gating mechanism. *Nat Commun* **12**, 2154 (2021).

2 Gao, Y., Cao, E., Julius, D. & Cheng, Y. TRPV1 structures in nanodiscs reveal mechanisms of ligand and lipid action. *Nature* **534**, 347-351 (2016).

3 Pumroy, R. A. *et al.* Molecular mechanism of TRPV2 channel modulation by cannabidiol. *Elife* **8**, e48792 (2019).

4 Zubcevic, L., Le, S., Yang, H. & Lee, S. Y. Conformational plasticity in the selectivity filter of the TRPV2 ion channel. *Nat Struct Mol Biol* **25**, 405-415 (2018).

5 Conde, J. *et al.* Allosteric Antagonist Modulation of TRPV2 by Piperlongumine Impairs Glioblastoma Progression. *ACS Cent Sci* **7**, 868-881 (2021).

6 Singh, A. K., McGoldrick, L. L. & Sobolevsky, A. I. Structure and gating mechanism of the transient receptor potential channel TRPV3. *Nat Struct Mol Biol* **25**, 805-813 (2018).

7 Neuberger, A., Nadezhdin, K. D., Zakharian, E. & Sobolevsky, A. I. Structural mechanism of TRPV3 channel inhibition by the plant-derived coumarin osthole. *EMBO Rep*, e53233 (2021).

8 Deng, Z. *et al.* Cryo-EM and X-ray structures of TRPV4 reveal insight into ion permeation and gating mechanisms. *Nat Struct Mol Biol* **25**, 252-260 (2018).

9 Hughes, T. E. *et al.* Structure-based characterization of novel TRPV5 inhibitors. *Elife* **8**, e49572 (2019).

10 Hughes, T. E. T. *et al.* Structural basis of TRPV5 channel inhibition by econazole revealed by cryo-EM. *Nat Struct Mol Biol* **25**, 53-60 (2018).

11 Dang, S. *et al.* Structural insight into TRPV5 channel function and modulation. *Proc Natl Acad Sci U S A* **116**, 8869-8878 (2019).

12 Singh, A. K., Saotome, K. & Sobolevsky, A. I. Swapping of transmembrane domains in the epithelial calcium channel TRPV6. *Sci Rep* **7**, 10669 (2017).

13 Singh, A. K., Saotome, K., McGoldrick, L. L. & Sobolevsky, A. I. Structural bases of TRP channel TRPV6 allosteric modulation by 2-APB. *Nat Commun* **9**, 2465 (2018).

14 Bhardwaj, R. *et al.* Inactivation-mimicking block of the epithelial calcium channel TRPV6. *Sci Adv* **6**, abe1508 (2020).

15 Singh, A. K., McGoldrick, L. L., Twomey, E. C. & Sobolevsky, A. I. Mechanism of calmodulin inactivation of the calcium-selective TRP channel TRPV6. *Sci Adv* **4**, eaau6088 (2018).
